# Supplementary material for: Dissecting the High Esterase/Lipase Activity and Probiotic Traits in Lactiplantibacillus plantarum B22: A Genome-Guided Functional Characterization
Source: Foods. 2025 Jul 2;14(13):2354. doi: 10.3390/foods14132354 (PMC12248764; doi:10.3390/foods14132354)
Supplement: Supplementary file 1 [file foods-14-02354-s001.zip › FigureS and TableS captions.pdf]

FigureS and TableS captions

Figure S1: Secondary structure prediction of *L. plantarum* B22 esterase/lipase enzymes;

Figure S2: Transmembrane region prediction of candidate esterase/lipase enzymes;

Figure S3: Multiple sequence alignment of *L. plantarum* B22 lipases with reference bacterial esterases;

Figure S4: Safety assessment of *L. plantarum* B22 for harmful metabolites;

Table S1: List of the selected 156 LAB isolates and the fermented food sources for each isolate;

Table S2: Lipid metabolism related pathways in *L. plantarum* B22 genome;

Table S3: Comparative analysis of antibiotic resistance genes for *L. plantarum* B22.
