# Supplementary material for: Dissecting the High Esterase/Lipase Activity and Probiotic Traits in Lactiplantibacillus plantarum B22: A Genome-Guided Functional Characterization
Source: Foods. 2025 Jul 2;14(13):2354. doi: 10.3390/foods14132354 (PMC12248764; doi:10.3390/foods14132354)
Supplement: Supplementary file 1 [file foods-14-02354-s001.zip › Table S1.pdf]

Table S1 List of the selected 156 LAB isolates and the fermented food sources for each isolate

Strain ID	Product Name	Origin	Strain ID	Product Name	Origin	Strain ID	Product Name	Origin
A05	Dryured beef	Kunming,Yunnan, China	A139-01	Goat milk	Honghe,Yunnan, China	B74-06	Schisandra wine	Diqing,Yunnan, China
A52-03	Yuxi Tonghai cured meat	Yuxi,Yunnan, China	A139-03	Goat milk	Honghe,Yunnan, China	B89	Horse feces	Diqing,Yunnan, China
A52-07	Yuxi Tonghai cured meat	Yuxi,Yunnan, China	B02-03	Black goat feces	Diqing,Yunnan, China	B97	Butter	Diqing,Yunnan, China
A52-09	Yuxi Tonghai cured meat	Yuxi,Yunnan, China	B02-07	Black goat feces	Diqing,Yunnan, China	B99-05	Butter	Diqing,Yunnan, China
A66-09	Pickled garlic	Yuxi,Yunnan, China	B03-04	Horse feces	Diqing,Yunnan, China	B99-02	Butter	Diqing,Yunnan, China
A66-14	Pickled garlic	Yuxi,Yunnan, China	B03-03	Horse fecess	Diqing,Yunnan, China	B102-06	Yak milk	Diqing,Yunnan, China
A70	Pickled garlic	Yuxi,Yunnan, China	B05-17	Pig feces	Diqing,Yunnan, China	B102-03	Yak milk	Diqing,Yunnan, China
A71	Cured pork	Yuxi,Yunnan, China	B005-04	Pig feces	Diqing,Yunnan, China	B116-05	Sheep feces	Dali,Yunnan, China
A72-10	Pickled radish	Yuxi,Yunnan, China	B05-07	Pig feces	Diqing,Yunnan, China	B116-2	Sheep feces	Dali,Yunnan, China
A72-13	Pickled radish	Yuxi,Yunnan, China	B05-08	Pig feces	Diqing,Yunnan, China	B116-14	Sheep feces	Dali,Yunnan, China
A73-01	Sour bamboo shoots	Yuxi,Yunnan, China	B08-09	Yak dry-cured beef	Diqing,Yunnan, China	B118-03-3	Goat milk	Dali,Yunnan, China

A73-02	Sour bamboo shoots	Yuxi,Yunnan, China	B08-11	Yak dry-cured beef	Diqing,Yunnan, China	B118-05-3	Goat milk	Dali,Yunnan, China
A73-04	Sour bamboo shoots	Yuxi,Yunnan, China	B008-14	Yak dry-cured beef	Diqing,Yunnan, China	B118-05	Goat milk	Dali,Yunnan, China
A74-07	Pckled radish	Yuxi,Yunnan, China	B17-04	Fresh butter	Diqing,Yunnan, China	B124	Sheep feces	Dali,Yunnan, China
A75	Pckled radish	Yuxi,Yunnan, China	B17-09	Fresh butter	Diqing,Yunnan, China	B127-01	Dairy cow feces	Dali,Yunnan, China
A76-01	Pickled vegetables	Yuxi,Yunnan, China	B17-11	Fresh butter	Diqing,Yunnan, China	B127-04	Dairy cow feces	Dali,Yunnan, China
A76-02	Pickled vegetables	Yuxi,Yunnan, China	B17-02	Fresh butter	Diqing,Yunnan, China	B130-05	Dairy cow feces	Dali,Yunnan, China
A76-03	Pickled vegetables	Yuxi,Yunnan, China	B22	Goat milk	Diqing,Yunnan, China	B130-09	Dairy cow feces	Dali,Yunnan, China
A76-07	Pickled vegetables	Yuxi,Yunnan, China	B24-06	Cured pork belly	Diqing,Yunnan, China	B135	Dairy cow feces	Dali,Yunnan, China
A77	Sauerkraut	Yuxi,Yunnan, China	B24-10	Cured pork belly	Diqing,Yunnan, China	B136	Black goat feces	Dali,Yunnan, China
A78	Qutuo sweet rice wine	Yuxi,Yunnan, China	B24-10-1	Cured pork belly	Diqing,Yunnan, China	B137-09	Goat milk	Dali,Yunnan, China
A79-11	Xiushan sweet fermented rice	Yuxi,Yunnan, China	B24-11	Cured pork belly	Diqing,Yunnan, China	B137-04-1	Goat milk	Dali,Yunnan, China
A79-02	Xiushan sweet fermented rice	Yuxi,Yunnan, China	B31-01	Butter	Diqing,Yunnan, China	B137-06	Goat milk	Dali,Yunnan, China
A79-04	Xiushan sweet fermented rice	Yuxi,Yunnan, China	B31-06	Butter	Diqing,Yunnan, China	B137-07	Goat milk	Dali,Yunnan, China

A79-07	Xiushan sweet fermented rice	Yuxi,Yunnan, China	B31-08	Butter	Diqing,Yunnan, China	B146-02	Black goat feces	Dali,Yunnan, China
A83-05	Rubing	Yuxi,Yunnan, China	B35-09	Fresh cheese curds	Diqing,Yunnan, China	B146-08	Black goat feces	Dali,Yunnan, China
A83-10	Rubing	Yuxi,Yunnan, China	B35-13	Fresh cheese curds	Diqing,Yunnan, China	B146-09	Black goat feces	Dali,Yunnan, China
A93-07-3	Cow milk	Honghe,Yunnan, China	B35-06	Fresh cheese curds	Diqing,Yunnan, China	B146-03	Black goat feces	Dali,Yunnan, China
A93-12	Cow milk	Honghe,Yunnan, China	B38-03	Pipa meat	Diqing,Yunnan, China	B146-04	Black goat feces	Dali,Yunnan, China
A119-13	Fresh milk	Kunming,Yunnan, China	B38-04	Pipa meat	Diqing,Yunnan, China	B150-02	Tinospora cordifolia stems	Dali,Yunnan, China
A119-03	Fresh milk	Kunming,Yunnan, China	B38-06	Pipa meat	Diqing,Yunnan, China	B150-06	Tinospora cordifolia stems	Dali,Yunnan, China
A119-07	Fresh milk	Kunming,Yunnan, China	B40	Pig feces	Diqing,Yunnan, China	B150-10	Tinospora cordifolia stems	Dali,Yunnan, China
A123-02	Xuanwei ham	Xuanwei,Yunnan, China	B44-04	Sheep feces	Diqing,Yunnan, China	B157-03	Tinospora cordifolia stems	Dali,Yunnan, China
A123-10S	Xuanwei ham	Xuanwei,Yunnan, China	B44-09	Sheep feces	Diqing,Yunnan, China	B157-11	Poshubaba	Dali,Yunnan, China
A123-17	Xuanwei ham	Xuanwei,Yunnan, China	B44-06-1	Sheep feces	Diqing,Yunnan, China	B157-01-3	Poshubaba	Dali,Yunnan, China
A123-02-1	Xuanwei ham	Xuanwei,Yunnan, China	B44-11	Sheep feces	Diqing,Yunnan, China	C15	Poshubaba	Dali,Yunnan, China
A124-03	Xuanwei ham	Xuanwei,Yunnan, China	B44-13	Sheep feces	Diqing,Yunnan, China	C25	Blood sausage	Dali,Yunnan, China

A131-05	Fuyuan ham	Qujing,Yunnan, China	B44-14	Sheep feces	Diqing,Yunnan, China	C29	Pork offal product	Dali,Yunnan, China
A133-01	Fuyuan ham	Qujing,Yunnan, China	B45-04	Yak milk	Diqing,Yunnan, China	C31	Goat cheese whey	Dali,Yunnan, China
A133-02	Fuyuan ham	Qujing,Yunnan, China	B45-07	Yak milk	Diqing,Yunnan, China	C39-09	Periplaneta americana (adult)	Dali,Yunnan, China
A133-06	Fuyuan ham	Qujing,Yunnan, China	B46	Yak feces	Diqing,Yunnan, China	C039-10	Periplaneta americana (adult)	Dali,Yunnan, China
A133-10	Fuyuan ham	Qujing,Yunnan, China	B48	Sour cow whey	Diqing,Yunnan, China	C39-13	Periplaneta americana (adult)	Dali,Yunnan, China
A134-02	Fuyuan pickled cabbage	Qujing,Yunnan, China	B50-05	Human breast milk	Diqing,Yunnan, China	C39-07	Periplaneta americana (adult)	Dali,Yunnan, China
A135-02	Fuyuan pickled radish	Qujing,Yunnan, China	B50-11	Human breast milk	Diqing,Yunnan, China	C41	Periplaneta americana (larvae)	Dali,Yunnan, China
A135-05	Fuyuan pickled radish	Qujing,Yunnan, China	B50-04	Human breast milk	Diqing,Yunnan, China	C68-01	Fermented chili	Dali,Yunnan, China
A136-01	Pickled radish	Qujing,Yunnan, China	B52-01	Hybrid cow-yak milk	Diqing,Yunnan, China	C68-02	Fermented chili	Dali,Yunnan, China
A136-07	Pickled radish	Qujing,Yunnan, China	B52-02	Hybrid cow-yak milk	Diqing,Yunnan, China	C74-10S	Papaya vinegar	Dali,Yunnan, China
A137-06	Fuyuan pickled cabbage	Qujing,Yunnan, China	B52-04	Hybrid cow-yak milk	Diqing,Yunnan, China	C74-13	Papaya vinegar	Dali,Yunnan, China
A137-15	Fuyuan pickled cabbage	Qujing,Yunnan, China	B61	Dried cheese sticks	Diqing,Yunnan, China	C106-4	Cow feces	Dali,Yunnan, China
A138-01	Fuyuan pickled cabbage	Qujing,Yunnan, China	B72	Erkuai (rice cake)	Diqing,Yunnan, China	C106-04	Cow feces	Dali,Yunnan, China

A138-07	Fuyuan pickled cabbage	Qujing,Yunnan, China	B74-05	Schisandra wine	Diqing,Yunnan, China	C106-08	Cow feces	Dali,Yunnan, China
B74-07	Schisandra wine	Diqing,Yunnan, China	B74-09	Schisandra wine	Diqing,Yunnan, China	C106-09	Cow feces	Dali,Yunnan, China
