# Supplementary material for: Dissecting the High Esterase/Lipase Activity and Probiotic Traits in Lactiplantibacillus plantarum B22: A Genome-Guided Functional Characterization
Source: Foods. 2025 Jul 2;14(13):2354. doi: 10.3390/foods14132354 (PMC12248764; doi:10.3390/foods14132354)
Supplement: Supplementary file 1 [file foods-14-02354-s001.zip › Table S2.pdf]

Table S2 Lipid metabolism related pathways in *L. plantarum* B22 genome

Gene ID	Gene Name	Description
**Glycerolipid metabolism**		
gene0157	-	triose/dihydroxyacetone kinase / FAD-AMP lyase (cyclizing) [EC:2.7.1.28 2.7.1.29 4.6.1.15]
gene0158	*dhaK*	phosphoenolpyruvate---glycerone phosphotransferase subunit DhaK [EC:2.7.1.121]
gene0159	*dhaL*	phosphoenolpyruvate---glycerone phosphotransferase subunit DhaL [EC:2.7.1.121]
gene0160	*dhaM*	phosphoenolpyruvate---glycerone phosphotransferase subunit DhaM [EC:2.7.1.121]
gene0175	*galA*	alpha-galactosidase [EC:3.2.1.22]
gene2997	*galA*	alpha-galactosidase [EC:3.2.1.22]
gene0345	*glpK*	glycerol kinase [EC:2.7.1.30]
gene0681	*glpK*	glycerol kinase [EC:2.7.1.30]
gene0942	*dagK*	diacylglycerol kinase (ATP) [EC:2.7.1.107]
gene1464	*dagK*	diacylglycerol kinase (ATP) [EC:2.7.1.107]
gene1897	*dagK*	diacylglycerol kinase (ATP) [EC:2.7.1.107]
gene1090	*bgsB*	1,2-diacylglycerol 3-alpha-glucosyltransferase [EC:2.4.1.337]
gene1096	*ltaS*	lipoteichoic acid synthase [EC:2.7.8.20]
gene2269	*ltaS*	lipoteichoic acid synthase [EC:2.7.8.20]
gene1397	*plsX*	phosphate acyltransferase [EC:2.3.1.274]
gene1569	*mgdA*	1,2-diacylglycerol 3-beta-glucosyltransferase [EC:2.4.1.336]
gene1579	*plsY*	acyl phosphate:glycerol-3-phosphate acyltransferase [EC:2.3.1.275]
gene1753	*plsC*	1-acyl-sn-glycerol-3-phosphate acyltransferase [EC:2.3.1.51]
gene2657	*dhaT*	1,3-propanediol dehydrogenase [EC:1.1.1.202]
gene28	*glxK*	glycerate 2-kinase [EC:2.7.1.165]

25		
**Fatty acid biosynthesis**		
gene0149	*fabG*	3-oxoacyl-[acyl-carrier protein] reductase [EC:1.1.1.100]
gene1434	*fabG*	3-oxoacyl-[acyl-carrier protein] reductase [EC:1.1.1.100]
gene1696	*fabG*	3-oxoacyl-[acyl-carrier protein] reductase [EC:1.1.1.100]
gene2413	*fabG*	3-oxoacyl-[acyl-carrier protein] reductase [EC:1.1.1.100]
gene0337	*accB*	acetyl-CoA carboxylase biotin carboxyl carrier protein
gene0522	*accB*	acetyl-CoA carboxylase biotin carboxyl carrier protein
gene1436	*accB*	acetyl-CoA carboxylase biotin carboxyl carrier protein
gene0521	*fabH*	3-oxoacyl-[acyl-carrier-protein] synthase III [EC:2.3.1.180]
gene1431	*fabH*	3-oxoacyl-[acyl-carrier-protein] synthase III [EC:2.3.1.180]
gene0523	*accC*	acetyl-CoA carboxylase, biotin carboxylase subunit [EC:6.4.1.2 6.3.4.14]
gene1438	*accC*	acetyl-CoA carboxylase, biotin carboxylase subunit [EC:6.4.1.2 6.3.4.14]
gene0524	*accD*	acetyl-CoA carboxylase carboxyl transferase subunit beta [EC:6.4.1.2 2.1.3.15]
gene1439	*accD*	acetyl-CoA carboxylase carboxyl transferase subunit beta [EC:6.4.1.2 2.1.3.15]
gene0525	*accA*	acetyl-CoA carboxylase carboxyl transferase subunit alpha [EC:6.4.1.2 2.1.3.15]
gene1440	*accA*	acetyl-CoA carboxylase carboxyl transferase subunit alpha [EC:6.4.1.2 2.1.3.15]
gene0574	-	fatty acyl-ACP thioesterase B [EC:3.1.2.14 3.1.2.21]
gene0744	*fabK*	enoyl-[acyl-carrier protein] reductase II [EC:1.3.1.9]
gene1430	*fabZ*	3-hydroxyacyl-[acyl-carrier-protein] dehydratase [EC:4.2.1.59]
gene1437	*fabZ*	3-hydroxyacyl-[acyl-carrier-protein] dehydratase [EC:4.2.1.59]
gene1433	*fabD*	[acyl-carrier-protein] S-malonyltransferase [EC:2.3.1.39]
gene1435	*fabF*	3-oxoacyl-[acyl-carrier-protein] synthase II [EC:2.3.1.179]

gene1441	*fabI*	enoyl-[acyl-carrier protein] reductase I [EC:1.3.1.9 1.3.1.10]
**Secondary bile acid biosynthesis**		
gene3034	*cbh*	choloylglycine hydrolase [EC:3.5.1.24]
**Sphingolipid metabolism**		
gene0175	*galA*	alpha-galactosidase [EC:3.2.1.22]
gene2997	*galA*	alpha-galactosidase [EC:3.2.1.22]
gene2995	*lacZ*	beta-galactosidase [EC:3.2.1.23]
gene2996	*lacZ*	beta-galactosidase [EC:3.2.1.23]
gene3132	-	hexosaminidase [EC:3.2.1.52]
**Fatty acid degradation**		
gene0089	*adh*	alcohol dehydrogenase [EC:1.1.1.1]
gene1426	*adh*	alcohol dehydrogenase [EC:1.1.1.1]
gene1666	*adh*	alcohol dehydrogenase [EC:1.1.1.1]
gene2341	*adh*	alcohol dehydrogenase [EC:1.1.1.1]
gene2505	*adh*	alcohol dehydrogenase [EC:1.1.1.1]
gene0304	*adhE*	acetaldehyde dehydrogenase / alcohol dehydrogenase [EC:1.2.1.10 1.1.1.1]
gene3148	*adhE*	acetaldehyde dehydrogenase / alcohol dehydrogenase [EC:1.2.1.10 1.1.1.1]
gene3067	*hcaD*	3-phenylpropionate/trans-cinnamate dioxygenase ferredoxin reductase component [EC:1.18.1.3]
**Primary bile acid biosynthesis**		
gene3034	*cbh*	choloylglycine hydrolase [EC:3.5.1.24]
**Glycerophospholipid metabolism**		
gene0244	*tagD*	glycerol-3-phosphate cytidylyltransferase [EC:2.7.7.39]
gene0998	*tagD*	glycerol-3-phosphate cytidylyltransferase [EC:2.7.7.39]
gene1068	*tagD*	glycerol-3-phosphate cytidylyltransferase [EC:2.7.7.39]
gene03	-	alpha-glycerophosphate oxidase [EC:1.1.3.21]

46		
gene0613	*gpsA*	glycerol-3-phosphate dehydrogenase (NAD(P)+) [EC:1.1.1.94]
gene0942	*dagK*	diacylglycerol kinase (ATP) [EC:2.7.1.107]
gene1464	*dagK*	diacylglycerol kinase (ATP) [EC:2.7.1.107]
gene1897	*dagK*	diacylglycerol kinase (ATP) [EC:2.7.1.107]
gene1145	*glpQ*	glycerophosphoryl diester phosphodiesterase [EC:3.1.4.46]
gene1350	*glpQ*	glycerophosphoryl diester phosphodiesterase [EC:3.1.4.46]
gene2073	*glpQ*	glycerophosphoryl diester phosphodiesterase [EC:3.1.4.46]
gene2270	*glpQ*	glycerophosphoryl diester phosphodiesterase [EC:3.1.4.46]
gene1579	*plsY*	acyl phosphate:glycerol-3-phosphate acyltransferase [EC:2.3.1.275]
gene1743	*cdsA*	phosphatidate cytidylyltransferase [EC:2.7.7.41]
gene1753	*plsC*	1-acyl-sn-glycerol-3-phosphate acyltransferase [EC:2.3.1.51]
gene2040	*pgsA*	CDP-diacylglycerol---glycerol-3-phosphate 3-phosphatidyltransferase [EC:2.7.8.5]
gene2832	*clsA_B*	cardiolipin synthase A/B [EC:2.7.8.-]
gene2845	*clsA_B*	cardiolipin synthase A/B [EC:2.7.8.-]
gene2899	*pgpA*	phosphatidylglycerophosphatase A [EC:3.1.3.27]
