# Supplementary material for: Dissecting the High Esterase/Lipase Activity and Probiotic Traits in Lactiplantibacillus plantarum B22: A Genome-Guided Functional Characterization
Source: Foods. 2025 Jul 2;14(13):2354. doi: 10.3390/foods14132354 (PMC12248764; doi:10.3390/foods14132354)
Supplement: Supplementary file 1 [file foods-14-02354-s001.zip › Table S3.pdf]

Table 3 Comparative analysis of antibiotic resistance genes for *L. plantarum* B22

Gene ID	Name	Drug Class
gene0083	*tet(T)*	tetracycline antibiotic
gene0098	*aadA27*	aminoglycoside antibiotic
gene0100	*macB*	macrolide antibiotic
gene0150	*bcrA*	peptide antibiotic
gene0201	*lin*	lincosamide antibiotic
gene0343	*mlaF*	oxazolidinone antibiotic
gene0390	*tetA(60)*	tetracycline antibiotic
gene0421	*Erm(K)*	lincosamide antibiotic;macrolide antibiotic;streptogramin antibiotic
gene0586	*optrA*	oxazolidinone antibiotic;phenicol antibiotic
gene0841	*rpsJ*	tetracycline antibiotic
gene0876	*novA*	aminocoumarin antibiotic
gene1252	*oleC*	macrolide antibiotic
gene1386	*arnA*	peptide antibiotic
gene1594	*tcr3*	tetracycline antibiotic
gene1643	*lmrB*	lincosamide antibiotic
gene1675	*MexR*	aminocoumarin antibiotic;carbapenem;cephalosporin;cephamycin;diaminopyrimidine antibiotic;fluoroquinolone antibiotic;macrolide antibiotic;monobactam;penam;penem;peptide antibiotic;phenicol antibiotic;sulfonamide antibiotic;tetracycline antibiotic
gene2111	*patB*	fluoroquinolone antibiotic
gene2213	*baeR*	aminocoumarin antibiotic;aminoglycoside antibiotic
gene2295	*lsaA*	lincosamide antibiotic;pleuromutilin antibiotic;streptogramin A antibiotic;streptogramin B antibiotic;streptogramin antibiotic
gene2521	*efpA*	isoniazid-like antibiotic;rifamycin antibiotic
gene2648	*patA*	fluoroquinolone antibiotic
gene2726	*golS*	carbapenem;cephalosporin;cephamycin;monobactam;penam;penem;phenicol antibiotic
gene2937	*bacA*	peptide antibiotic
gene3062	*rosB*	peptide antibiotic
