# Supplementary figures and images for: Dissecting the High Esterase/Lipase Activity and Probiotic Traits in Lactiplantibacillus plantarum B22: A Genome-Guided Functional Characterization
Source: Foods. 2025 Jul 2;14(13):2354. doi: 10.3390/foods14132354 (PMC12248764; doi:10.3390/foods14132354)

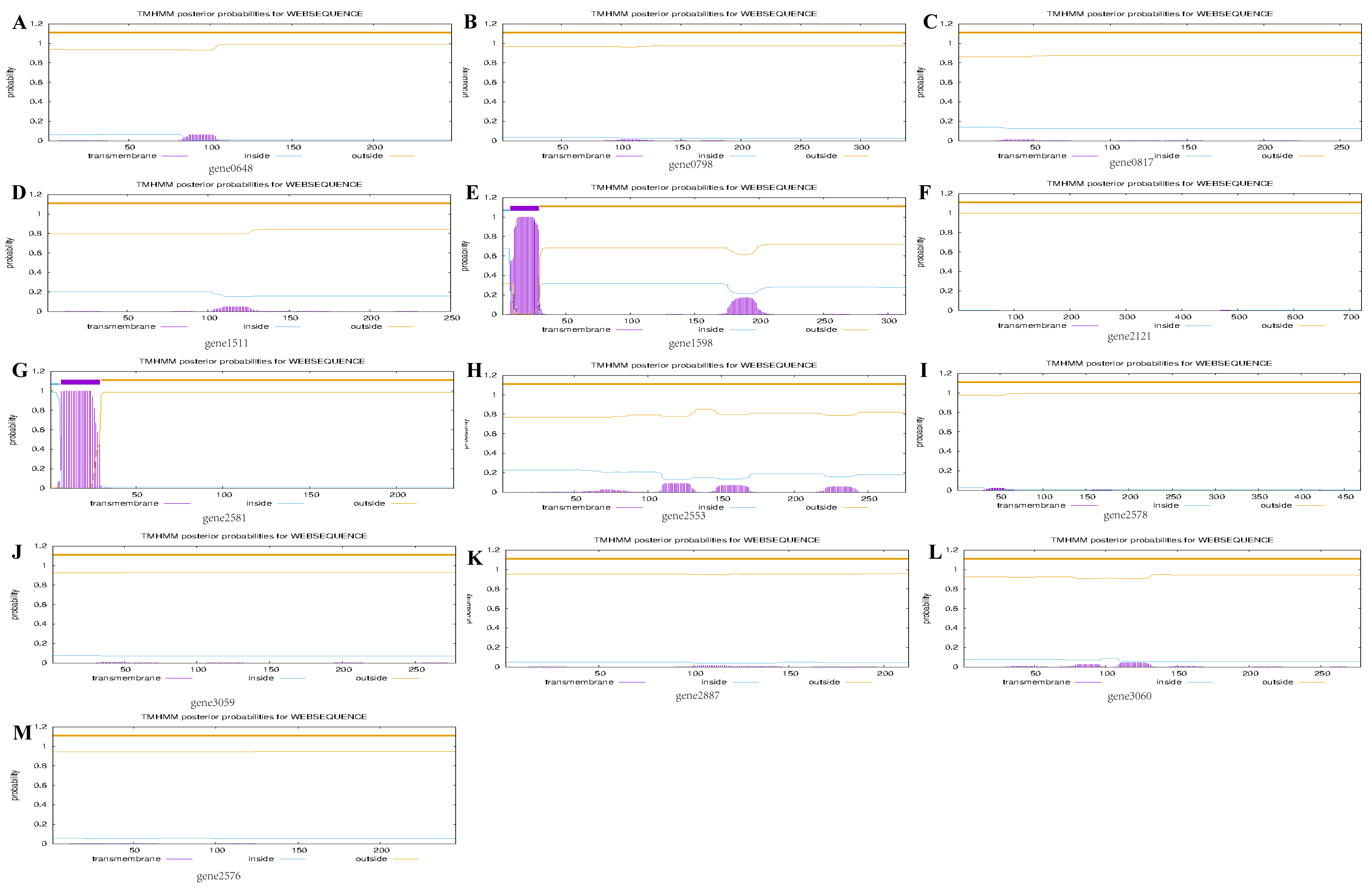

Supplement: Supplementary file 1 [file foods-14-02354-s001.zip › Fig.S1.tif]

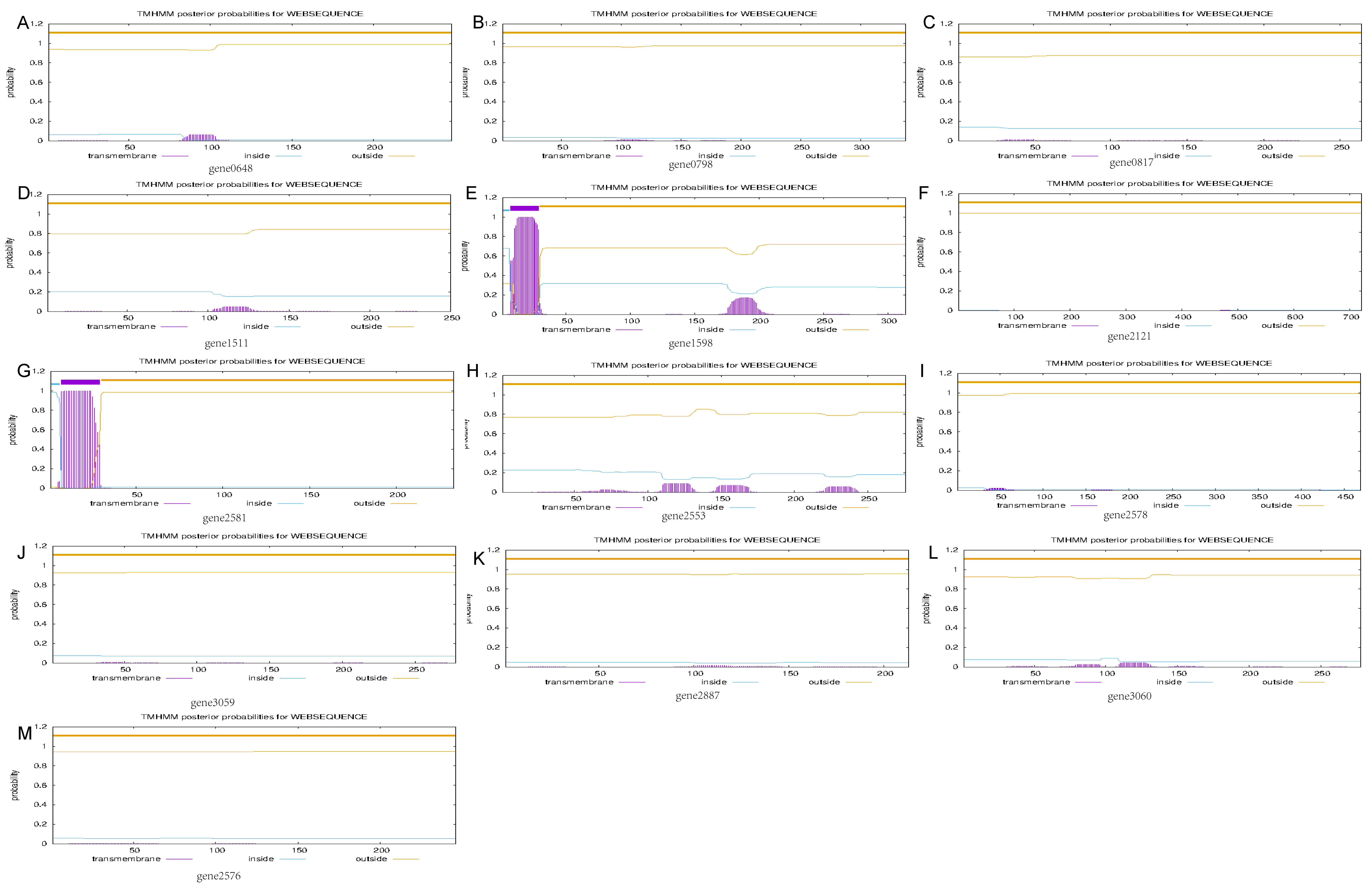

Supplement: Supplementary file 1 [file foods-14-02354-s001.zip › Fig.S2.tif]

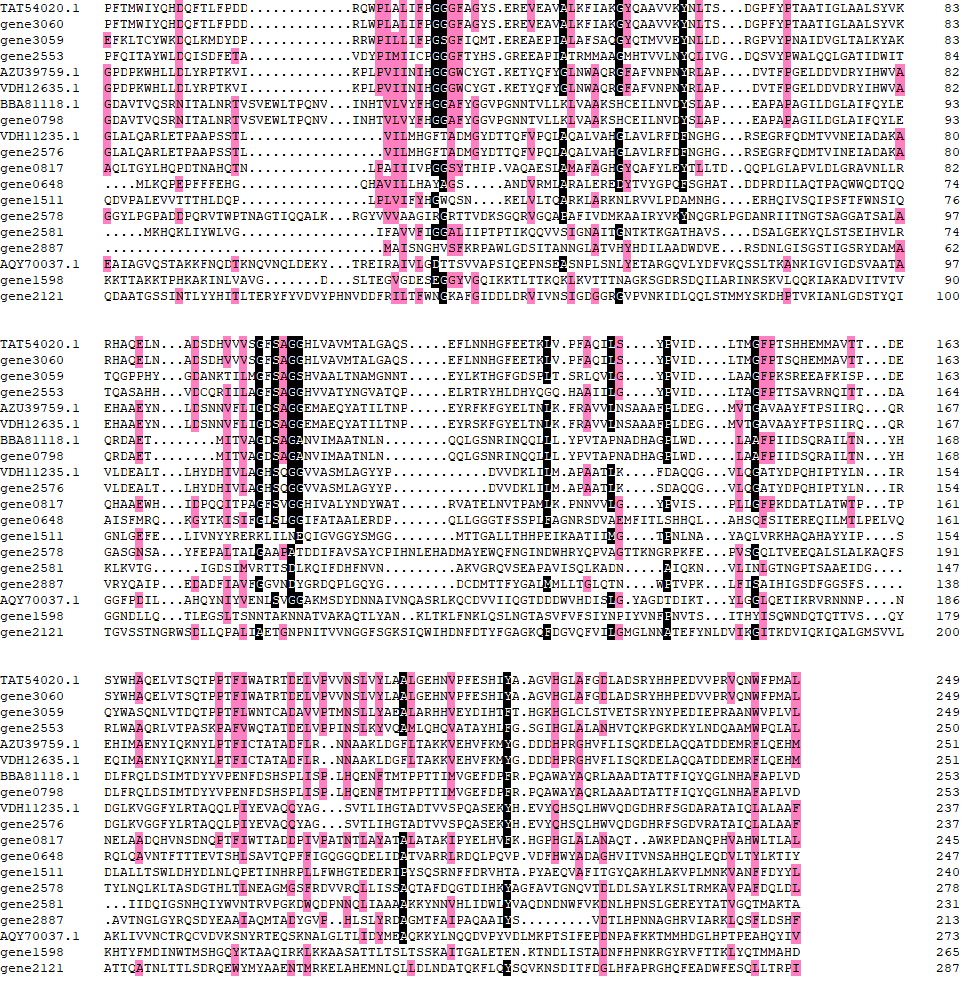

Supplement: Supplementary file 1 [file foods-14-02354-s001.zip › Fig.S3.tif]

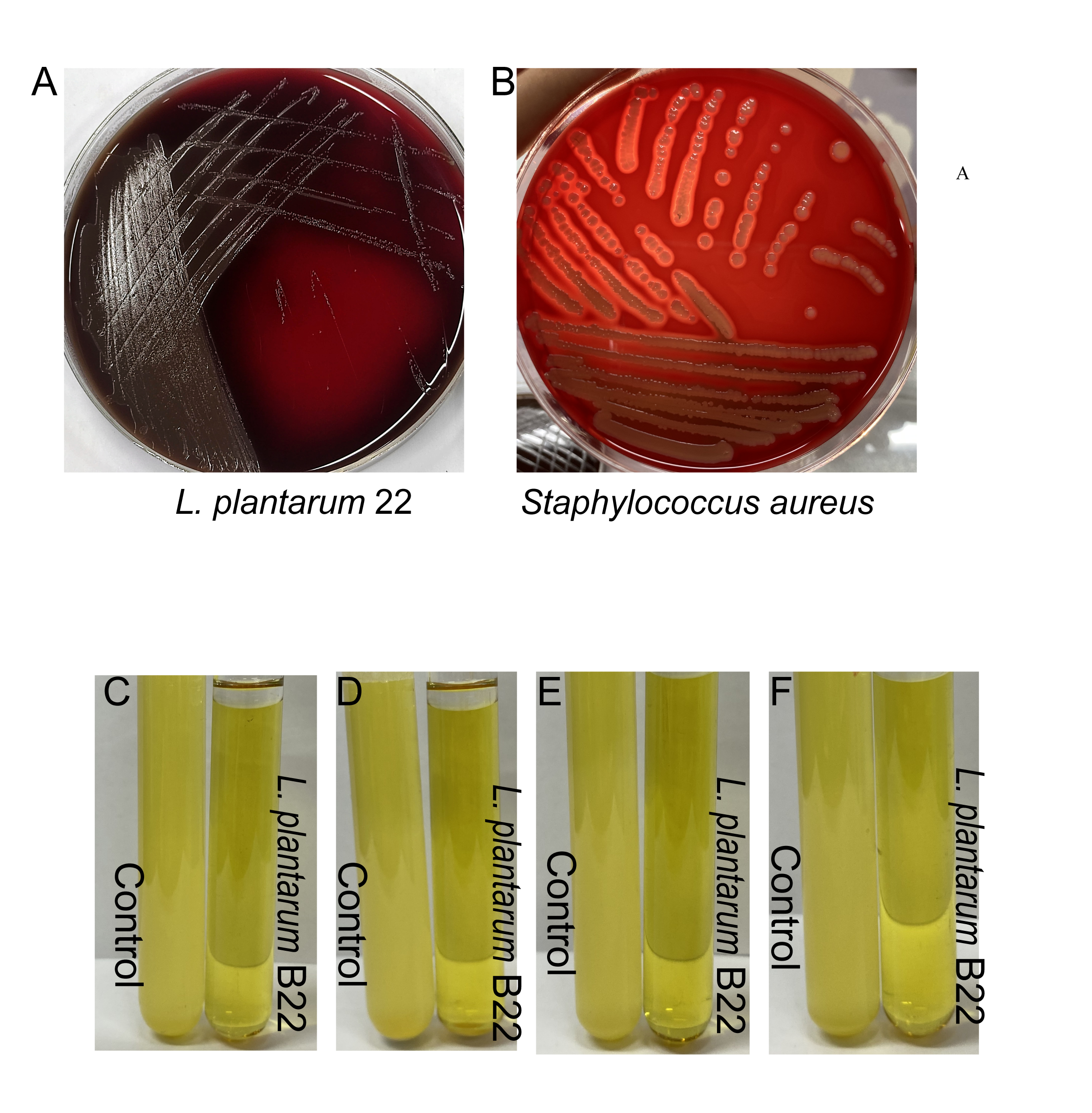

Supplement: Supplementary file 1 [file foods-14-02354-s001.zip › Fig.S4.tif]
